# Supplementary material for: Pituitary tissue-specific miR-7a-5p regulates FSH expression in rat anterior adenohypophyseal cells
Source: PeerJ. 2019 Apr 9;7:e6458. doi: 10.7717/peerj.6458 (PMC6461031; doi:10.7717/peerj.6458)
Supplement: Supplemental Information 1 [file peerj-07-6458-s001.docx]

**S1 File. Construction of pmiR-FSHb-3’UTR-MUT reporter plasmid**

The full-length 3’UTR of rat FSHb mRNA was cloned between the XhoI and NotI sites of the pmiR-RB-REPORT^TM^ plasmid. To disrupt the binding site of the FSHb 3’UTR, the target sequence TCTTCCA (574-580) was mutated into AGAAGGT, forming the pmiR-FSHb-3’UTR-MUT plasmid. The primers used in the colony experiment were as follows:

FSHb F: GCGCTCGAGGGAACAATGGACATTGCC

FSHb R: AATGCGGCCGCTTCATCAGTACGACTTTA

FSHb-MUT F: TACTCAACAGAAGGTAAGCATGTGGAGTATTGA

FSHb-MUT R: ACATGCTTACCTTCTGTTGAGTATCCTAACCTT

The PCR colony was identified after purification of the PCR product, enzyme cleavage, purification of the cleavage product, connection and convention.

The plasmid was extracted from the colony, and the sequence was identified by a sequencing company. The results of sequencing (Fig 1) showed that the target sequences were mutated successfully.


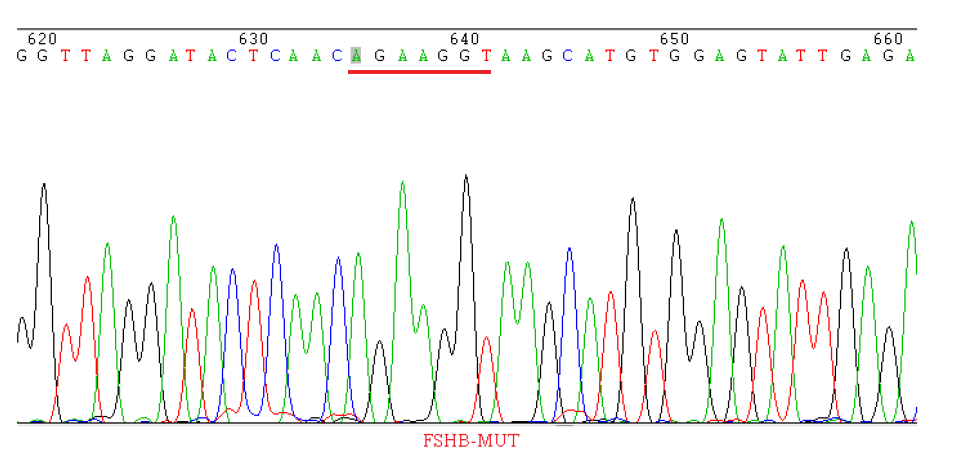


**S1. Fig 1. DNA sequence peak map.** Sequence of the extracted plasmid; the target sequence was mutated into AGAAGGT.
